# Ageism and People With Early-Onset Disability

**DOI:** 10.1093/geroni/igaa057.3107

**Published:** 2020-12-16

**Authors:** Patricia Heyn

**Affiliations:** University of Colorado Anschutz Medical Campus, Aurora, Colorado, United States

## Abstract

People with disabilities are underrepresented in health research and usually they are explicitly excluded from research participation. There are no longitudinal studies that follows cohorts with disabilities to learn about their healthspan and aging processes. Thus, we know very little about how people with early onset disability age, and the medical conditions and health risk factors contributing to the “accelerated aging” phenomena commonly observed in this population. Our research group has been following a cohort group of individuals with pediatric onset disability since 2013. We collected pediatric and adult data from 70 persons with cerebral palsy and we evaluated key health outcomes as well as their access to care. In this session, we will present some of the key findings from our longitudinal study. We will discuss future research and key areas that needs to be addressed for us to properly move forward this urgent unmet area of aging research.

